# Multiple large enteroliths associated with an incisional hernia: a rare case

**DOI:** 10.1308/003588412X13373405385890

**Published:** 2012-10

**Authors:** I Wilson, U Parampalli, C Butler, I Ahmed, A Mowat

**Affiliations:** Medway HS Foundation Trust,UK

**Keywords:** Enterolith, Fecalith, Hernia

## Abstract

The surgeon frequently encounters renal and biliary stones but rarely may also encounter enteric stones or enteroliths. An enterolith is a stony foreign body that is formed in the gastrointestinal tract. We present a rare case of multiple, large enteroliths found associated with a longstanding incarcerated incisional hernia.

## Case history

An 82-year-old woman presented with vomiting and a large, painful lump in the right iliac fossa. The surgical history was extensive with the patient having undergone a Caesarean section 42 years previously through a midline incision. Fourteen years later, she developed an incarcerated incisional hernia requiring a further laparotomy with small bowel resection. The patient developed further multiple hernias of the anterior abdominal wall along with an enterocutaneous fistula.

**Figure 1:**
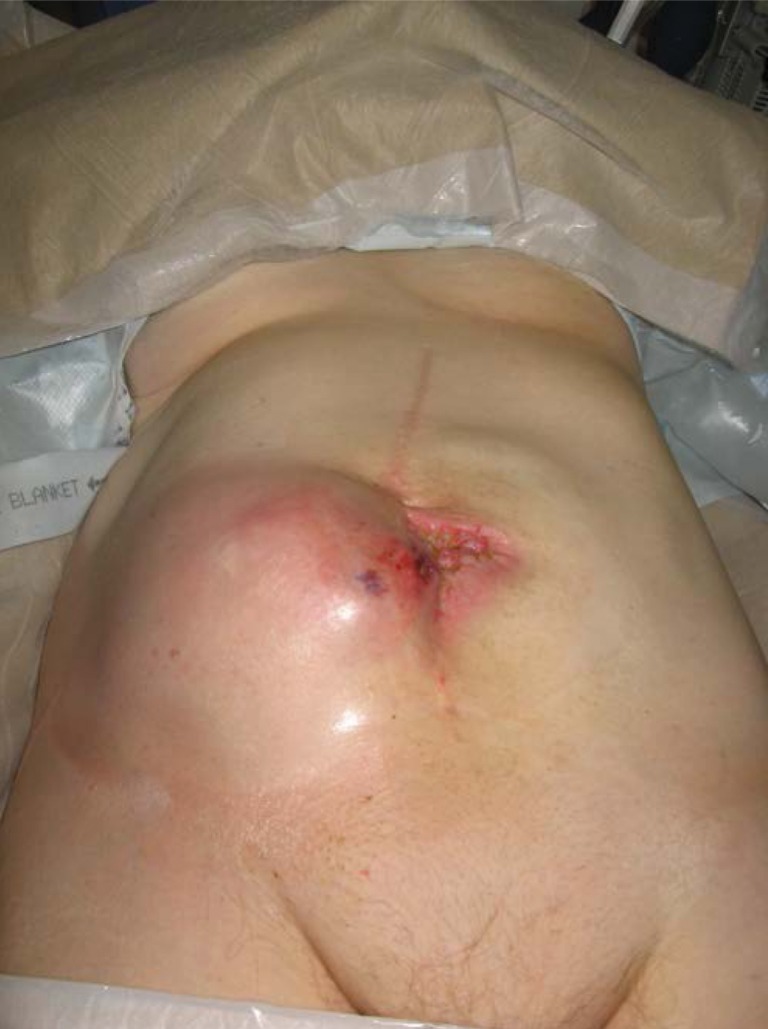
Patient prior to laparotomy showing the large incisional hernia in the right lower quadrant of the abdomen with midline enterocutaneous fistula

**Figure 2:**
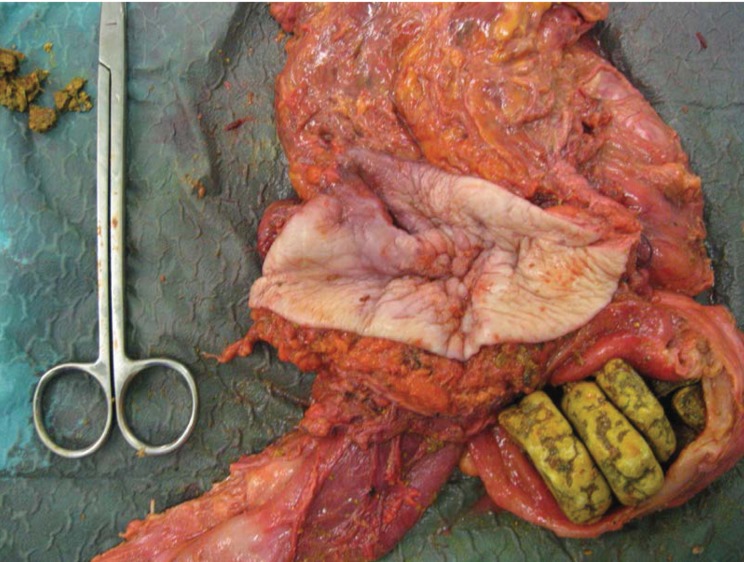
Contrast enhanced computed tomography of abdomen and pelvis: coronal (left) and transverse (middle) view showing enteroliths in the dilated loop of bowel in the abdomen and transverse view showing the large anterior abdominal wall hernia (right)

On presentation, abdominal examination revealed a 15cm × 15cm mass in the right lower quadrant (Fig 1). This mass was tender, hard and irreducible. Contrast enhanced computed tomography (CT) of the abdomen and pelvis revealed a large right-sided hernia containing caecum, with collapse of the proximal colon and dilatation of the distal ileum. A dilated loop of small bowel in the abdomen, 8cm in diameter and containing foreign bodies, was also noted (Fig 2).

A diagnosis of a strangulated incisional hernia with associated enterocutaneous fistula was made. A laparotomy revealed strangulated sections of terminal ileum, caecum and ascending colon in the right-sided incisional hernia. Several stony structures could be palpated in the distended terminal ileum, which was also found to be communicating with the skin via the enterocutaneous fistula. No gallstones could be palpated in the gallbladder and there was no fistulation between the bowel and biliary tree. *En bloc* resection of the caecum, enterocutaneous fistula and distended section of terminal ileum was undertaken. The abdominal wall defect was reconstructed using a biological mesh.

**Figure 3:**
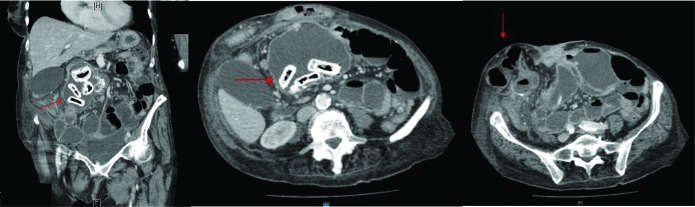
Resected specimen showing five enteroliths in the distended loop of terminal ileum

Inspection of the distended section of terminal ileum revealed five green, stony foreign bodies, the largest of which measured 4.7cm by 3.0cm. These foreign bodies were observed to have a concentric ring pattern (Fig 3). The appearance and consistency of these foreign bodies was felt to be consistent with true enteroliths. The enteroliths were sent for analysis in formalin. However, on submersion in the formalin, the enteroliths dissolved.

## Discussion

Foreign bodies are found throughout the gastrointestinal tract. These may be either exogenous, having been ingested, or endogenous if formed in the gastrointestinal tract. Enteroliths (endogenous stony foreign bodies or concretions) may be classified as being either true or false. False enteroliths such as faecaliths are formed from ingested and usually indigestible bowel contents. True enteroliths are concretions of insoluble precipitates of substances normally found with the gastrointestinal tract. There are two groups of true enteroliths: those formed mainly from bile acids and those composed mostly of mineral salts.

True enteroliths composed principally of bile salts can be subdivided further into primary and secondary. Primary enteroliths form in the bowel lumen whereas secondary enteroliths develop in the biliary tree and reach the bowel through either the ampulla of Vater or a fistula.^1^

Whether true or false, enteroliths are formed in areas of stagnation of bowel contents. False enteroliths (eg faecaliths) are formed from the prolonged accumulation and compaction of bowel contents in areas of poor gastrointestinal flow such as the appendix, a Meckel’s diverticulum or the chronically constipated bowel.^2^ With regards to true enteroliths, stasis in the gastrointestinal tract promotes local bacterial overgrowth similar to that observed in blind loop syndrome. Bacterial overgrowth leads to changes in pH of the surrounding bowel contents, resulting in precipitation of substances out of solution.^3^ The accumulation and concretion of these precipitates leads to formation of a true enterolith. Some authors have shown enteroliths form around an indigestible nidus^4^ although this is not found in all enteroliths.

Various substances have been found in enteroliths. True enteroliths are composed predominantly of bile acids or calcium salts. This is related to the pH of the bowel where enterolith formation takes place. Bile salts precipitate at a lower pH so enteroliths formed in the duodenum and jejunum are formed mostly from choleic acid. The more alkaline distal bowel, however, favours the formation of enteroliths composed of calcium salts, for example calcium oxalate.^1^ False enteroliths are made up of indigetable bowel contents including faeces (faecaliths), hair (trichobezoars) and vegetable matter (phytobezoars). It is worth noting that once a false enterolith has formed, calcium salts may be deposited in and around the false enterolith, giving rise to a structure that can resemble a true enterolith.^1^

In our case, the stony structures present in the distal ileum were faecaliths, a type of false enterolith, and not, as initially thought, true enteroliths. This case of false enteroliths is particularly unique due to the large size and number of the faecaliths. Furthermore, more significantly, the association of false enteroliths with a chronically incarcerated hernia and a persistent enterocutaneous fistula has, to our knowledge, not been reported previously.

In this patient, faecalith formation is likely to have occurred due to the stagnant flow created by the downstream chronically incarcerated bowel of the abdominal wall hernia. The large size and number of faecaliths can be explained by the fact that once the initial faecaliths had reached a certain size, they will themselves have caused stasis of the bowel, creating a favourable environment for faecalith growth and formation.^5^ This vicious circle of bowel stagnation will also have been the cause for the chronic enterocutaneous fistula. The calcific appearance of the faecaliths on CT and their stony texture on palpation may be explained by the theory that the sluggish flow created by both the incarcerated bowel and large faecaliths will have provided a favourable environment for bacterial overgrowth, which, similar to that observed in the formation of true enteroliths, will have led to calcium deposition in and around the faecaliths.

## Conclusions

It is important for the surgeon to have an understanding of the aetiology and classification of enteroliths. The surgeon must be aware that enteroliths are formed in areas of bowel stasis so surgical management should also aim to identify and correct the pathology that led to enterolith formation to avoid future enterolithiasis.
